# sCD163, sCD28, sCD80, and sCTLA-4 as soluble marker candidates for detecting immunosenescence

**DOI:** 10.1186/s12979-023-00405-0

**Published:** 2024-01-20

**Authors:** Andrea Aprilia, Kusworini Handono, Hidayat Sujuti, Akhmad Sabarudin, Nuning Winaris

**Affiliations:** 1 Doctoral Program in Medical Science, Faculty of Medicine, Universitas Brawijaya, Malang, Indonesia; 2 Clinical Pathology Department, Faculty of Medicine, Universitas Brawijaya, Veteran Street, Malang, East Java 65145 Indonesia; 3 Opthamology Department, Faculty of Medicine, Universitas Brawijaya, Malang, Indonesia; 4 Chemistry Department, Faculty of Mathematics and Science, Universitas Brawijaya, Malang, Indonesia; 5 Department of Parasitology, Faculty of Medicine, Universitas Brawijaya, Malang, Indonesia

**Keywords:** Immunosenescence, Aging, Biomarker, Soluble marker, Immune system

## Abstract

**Background:**

Inflammaging, the characteristics of immunosenescence, characterized by continuous chronic inflammation that could not be resolved. It is not only affect older people but can also occur in young individuals, especially those suffering from chronic inflammatory conditions such as autoimmune disease, malignancy, or chronic infection. This condition led to altered immune function and as consequent immune function is reduced. Detection of immunosenescence has been done by examining the immune risk profile (IRP), which uses flow cytometry. These tests are not always available in health facilities, especially in developing countries and require fresh whole blood samples. Therefore, it is necessary to find biomarkers that can be tested using stored serum to make it easier to refer to the examination. Here we proposed an insight for soluble biomarkers which represented immune cells activities and exhaustion, namely sCD163, sCD28, sCD80, and sCTLA-4. Those markers were reported to be elevated in chronic diseases that caused early aging and easily detected from serum samples using ELISA method, unlike IRP. Therefore, we conclude these soluble markers are beneficial to predict pathological condition of immunosenescence.

**Aim:**

To identify soluble biomarkers that could replace IRP for detecting immunosenescence.

**Conclusion:**

Soluble costimulatory molecule suchsCD163, sCD28, sCD80, and sCTLA-4 are potential biomarkers for detecting immunosenescence.

## Background

The concept of “inflammaging” introduced by Franceschi et al., (2000), was related to declining ability of immune system to cope with various external stressors, as well as accelerating pro-inflammatory status. They proposed that continuous exposure to internal and external stressors led to chronic macrophage activation, naïve immune cells exhaustion, decreased in the T-cell repertoire and affected other immune cells. These continuous disruptions over the time resulting a major change and depleted the ability of immune system to response against stressors, as well as escalating pro-inflammatory response. These conditions define the characteristic of immunosenescence [1].

Immunosenescence was commonly identified with immune risk profile (IRP). The concept of IRP has originated from the OCTO/NONA study conducted in Sweden in individuals over 85 years of age. IRP is an immunosenescence marker characterized by the presence of 100% cytomegalovirus (CMV) infection and a CD4/CD8 ratio < 1 owing to the accumulation of differentiated T cells, particularly CD27^−^CD28^−^CD57^−^CD8^+^ T cells. These are the antigen-specific T cells for CMV [2–4]. Furthermore, IRP is characterized by a lower number of CD8^+^CD45RA^+^ cells and higher number of CD8^+^CD45RO^+^ T cells [3, 5, 6].

An inverted CD4/CD8 ratio (< 1) is associated with an increase in the number of activated, senescent, and exhausted CD4^+^ T cells and CD8^+^ T cells, as well as a shift in naïve to memory cells [7]. In addition, an inverted CD4/CD8 ratio indicates accumulation of CD8^+^ T cells that have differentiated into the late phase, a low proliferative response of T cells, and a low number of B cells [5]. An inverted CD4/CD8 ratio has been linked with premature immunosenescence in individuals of all ages, including children and young adults infected with Human Immunodeficiency Virus (HIV), patients with myocardial infarction, and patients under physical and psychological stress. The prevalence of an inverted CD4/CD8 ratio is 8% between the age of 20 and 59 and up to 16% between the age of 60 and 94. The inverted CD4/CD8 ratio correlates with an increased mortality and persistent viral infection [8].

In addition to the inverted CD4/CD8 ratio, one of the IRPs is a decrease in T-cells with CD28 receptors. CD28 is a costimulatory molecule responsible for activating T cells. However, during activation, some T cells lose the CD28 molecule and become CD28^−^ T cells. These CD28^−^ T cells act as antigen-recognizing cells and are highly differentiated. During normal aging, CD8^+^CD28^−^ T cells are accumulated, which is likely due to the continuous exposure to various antigens present through lifetime [9]. A study by Yadav et al. found that CD4^+^CD28^−^ T cells in patients with chronic kidney disease (CKD) were correlated with an increased occurrence of atherosclerosis [10]. CD4^+^CD28^−^ T cells also show increased cytotoxic and inflammatory activity. Téo et al. showed that CD4^+^CD28^−^ T cells participate in the pathogenesis of atherosclerosis and their number also increases in patients with acute coronary syndrome (ACS) [11].

An increase in memory T cells (CD4^+^CD45RO^+^ T cells and CD8^+^CD45RO^+^ T cells) and a decrease in naïve T cells (CD4^+^CD45RA^+^ T cells and CD8^+^CD45RA^+^ T cells) is observed during immunosenescence. Memory T cells increase with age and are more abundant in tissues [12]. Naïve T cells (CD45RA^+^) express CD27, CD28, and CCR7 as they leave the thymus. When exposed to antigens, naïve T cells differentiate into central memory T cells (CD45RO^+^ T cells) [13]. Involution of the thymus leads to a decrease in naïve T cells. These conditions make geriatric population more susceptible to new antigens [14].

In addition to these parameters, IRP is also characterized by the presence of CMV infection. CMV mainly survives in myeloid cells but can sometimes be found in other cells. When CMV infection occurs, containment of the wider infection is a priority for the immune system; however, complete elimination is never achieved. A study found changes in CD8^+^ T cells that were very similar to senescence but occurred as a result of CMV infection [15]. The CMV seropositive parameter can predict mortality in the geriatric population. These findings indicate the presence of persistent CMV as a chronic antigen stressor, which is a major contributor to immunosenescence and mortality [15, 16].

Although many IRPs have been identified, which can be used for the detection of immunosenescence, these markers are cell surface receptors that have to be examined using flow cytometry, which required a fresh blood sample. The required condition is difficult to accommodate especially in developing countries, where most of the healthcare facilities lack of advance instruments, such as flow cytometry. As a result, immunosenescence markers in the dissolved form are required. Markers in the dissolved form are more stable and could be measured from stored serum. Other promising soluble marker candidates for accelerated aging, such as soluble urokinase plasminogen activator receptor (suPAR) was not easily detected, as it was present in low concentration in the serum [17]. Other soluble markers such as IL-6, TNF-α and IFN-γ were also commonly used as biomarkers for immunosenescence detection, however those markers were less specific as it can be also detected during acute inflammation [18–20].

This review focused on four different soluble markers, CD163, CD28, CD80 and CTLA-4, which were commonly known to have various functions related to macrophage [21–23] and T cells activation [24–26], which play a key role in immunosenescence. Characterization of CD163 and CD80 biomarkers, which determine the macrophage polarization, had been used as to monitor inflammaging [27].

While CD28 is linked to inflammaging as it is observed to be absent in elder people as well as age-related diseases. The senescence is associated with an increase of CD28^-^ memory T cell and decrease of naïve T cell populations, therefore the CD28 could be a good biomarker candidate of immunosenescence [28]. CD28^-^ T cells are also known to have a short telomerase, as a consequence it will affect negatively on the immune checkpoint inhibitory receptors, such as CTLA-4. Suppressive function of Treg was regulated by CTLA-4 receptor together with the co-stimulatory receptor CD28. Therefore, CTLA-4, together with PD-1, which regulate the T cells response, play important roles in maintaining the balance between stimulatory and inhibitory signals for immune responses against antigens [28, 29], thus determine the senescence status.

Moreover, the soluble markers mentioned above were easily detected using enzyme-linked immunosorbent assay (ELISA) method, as they were present in high concentration in the serum [30–33].

### *Soluble* CD163

CD163 is a glycosylated membrane protein [23] expressed almost exclusively on all macrophages [21–23] and at least 10-30% of monocytes [34]. CD163 is a member of the B scavenger receptor cysteine-rich (SRCR) family [22] and consists of 9 extracellular SRCR protein domains associated with the short transmembrane segment and short cytoplasmic tail. CD163 is located on chromosome 12p13 and consists of 17 exons [23]. CD163 is also a scavenger hemoglobin (Hb) receptor involved in the endocytosis of the haptoglobin-Hb complex [21, 23]. It has other functions as well, including those related to erythroblast adhesion, immune sensing of the presence of bacteria, and bonding with TNF-like weak inducer of apoptosis (TWEAK) [23]. In vitro, CD163 expression is increased by the presence of glucocorticoids, IL-10 and IL-6 but not by IL-4 or IL-13, but not by IL-4 and IL-13 [21, 23]. CD163 expression is reduced by the presence of tumors necrosis factor-alpha (TNF-α), interferon-gamma (IFN-γ), and chemokine CXCL4 (platelet factor 4). This suggests that CD163 is mainly expressed by M2 macrophages [23].

CD163 can be released (shedding) upon the action of cleaving enzyme TNF-α [35], resulting in a dissolved form known as soluble CD163 (sCD163) [23, 35]. The sCD163 level is negatively correlated with the level of membrane bound CD163 [35]. sCD163 has been detected in the plasma of healthy individuals at levels ranging from 0.73 to 4.69 mg/L, with a median of 1.87 mg/L [36]. Because its expression is limited to the monocyte line, sCD163 can be used as a specific marker of macrophage and monocyte activation [22, 35]. Furthermore, sCD163 and non-membrane-bound products function similarly to cytokines in that they inhibit the activation and proliferation of T lymphocytes [35], particularly CD4^+^ T cells [36]. The release of sCD163 by cleaving enzyme TNF-α corresponds to an increase in sCD163 levels in acute and chronic inflammatory diseases [35, 37] and hematological diseases [35].

**Fig. 1:**
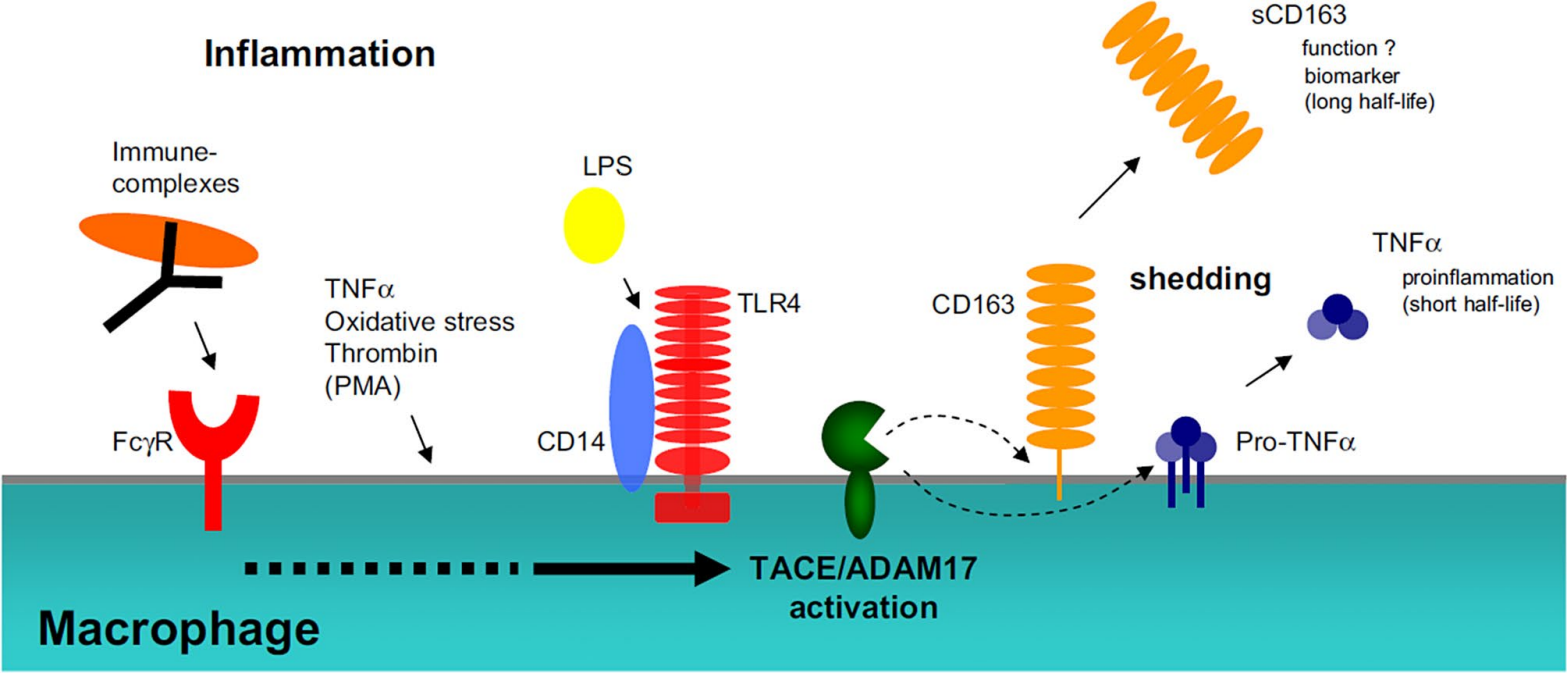
Shedding of soluble CD163. Various inflammatory signals induce shedding of sCD163 in vitro. Shedding of sCD163 can be induced by TLR4 activation or FcγR-crosslinking mediated by ADAM17/TACE. Half-life of sCD163 is longer than that of TNF-α. FcγR, Fc-gamma receptors; LPS, Lipopolysaccharides; TACE/ADAM17, Tumor necrosis factor α-converting enzyme; TLR, Toll-like receptors; TNF, Tumor necrosis factor. Reprinted with permission from Reference: Møller, H. J. 2012. Soluble CD163. *Scandinavian journal of clinical and laboratory investigation*, 72, 1–13

In vivo, the release of sCD163 occurred simultaneously with an increase in TNF-α response due to the presence of lipopolysaccharide (LPS) mediated by Toll-like Receptors (TLR) 4 activation. Then, it is accompanied by a sharp increase in sCD163 and TNF-α levels and positively correlated with the number and activity of monocytes or macrophages (Fig. 1) [23, 38]. In addition, sCD163 was released in the presence of oxidants and pro-inflammatory cytokines [39]. The half-life of sCD163 is much longer than TNF-α and sCD163 levels remain elevated for 1–2 days [23, 40]. Levels of sCD163 can be affected by several factors such as increased CD163 expression, increased shedding, and impaired clearance [23]. In healthy individuals, sCD163 levels were shown to have low individual variability with a value limit of ± 30% [35].

Various studies have been conducted to better understand sCD163; however, research on sCD163 in the context of immunosenescence in geriatrics is lacking. Previous studies have shown that sCD163 is elevated in patients with HIV and is linked with the aging of CD8^+^ T cells in these patients. Researchers have discovered that sCD163 levels in patients with HIV were equivalent to those of 14.5 year older healthy individuals. Furthermore, an increase in sCD163 with age has been shown [41]. Other HIV-related studies have demonstrated a negative relationship between sCD163 levels and telomere length. During HIV infection, telomere shortening occurs, which is a process associated with aging. Increased sCD163 in patients with HIV infection also correlates with the emergence of co-morbidities in the form of premature degenerative diseases such as cardiovascular and neurological diseases [42, 43]. This indicates a correlation between chronic immune system activation and increased sCD163 levels.

Hodowanec et al. found that sCD163 levels positively correlated with anti-CMV IgG antibody levels were positively correlated in patients with HIV infection [44]. Azanan et al. found an increase in sCD163 in pediatric patients with leukemia when compared with healthy children of the same age; sCD163 levels correlated with anti-CMV IgG antibodies in these patients [45]. Another study found an increase in sCD163 in patients with chronic Hepatitis C Virus (HCV) infection. sCD163 is also associated with increased mortality from heart disease, acute myocardial infarction, increased mortality, atherosclerosis, diabetes, and insulin resistance [46].

Various studies regarding sCD163 levels in serum and urine samples of various chronic diseases are listed in Table 1. In addition, Zhi et al. analyzed the role of sCD163 in asthma. According to their study, sCD163 plays a role in the pathogenesis of asthma and can act as a potential marker as well as a target for therapy [47]. Meanwhile, the meta-analysis by Qian et al. suggested that sCD163 is correlated with the risk of mortality in cancer [48].

Based on Table 1, it can be concluded that sCD163 levels, especially serum sCD163, correlate with various chronic inflammatory conditions arising due to infection, autoimmunity, or malignancy. Chronic inflammatory conditions are similar to inflammaging in immunosenescence. However, to the best of our knowledge, no studies have reported the role of sCD163 in immunosenescence.

**Table 1:** The role of sCD163 in various chronic diseases

Disease	sCD163 level (specimen)	Clinical Importance	Ref
Atherosclerosis	2.469 (0.264–9.063) mg/L (plasma)	Elevated in coronary atherosclerosis.	[49]
Liver failure	808.6 ± 433.0 ng/mL (serum)	Elevated in fulminant liver failure, positively correlated with prolonged prothrombin time and mortality.	[50]
Cirrhosis	4.5 mg/L (plasma)	Elevated in cirrhosis and has positive correlation with Child-Pugh classification, also portal hypertension predictor marker.	[51]
	5.77 mg/L (plasma)	Elevated in cirrhosis that caused by Hepatitis C Virus (HCV) and correlated with other inflammatory markers.	[52]
*Non-alcoholic fatty liver* *disease* (NAFLD)	2.5–3.9 mg/L (plasma)	Liver fibrosis predictor.	[53]
Type 2 Diabetes Mellitus (T2DM)	1.95 (0.63–6.97) mg/L (serum)	Elevated in T2DM and has positive correlation with insulin resistance.	[54]
Obesity in chronic kidney disease (CKD) stage V	4.0 mg/L (plasma)	Has positive correlation with increased fat mass and other inflammatory markers in CKD stage V.	[55]
HIV infection	2.89 (2.22–3.42) mg/L (plasma)	Correlated with RNA viral load, risk for cardiovascular event (age, ethnic, body mass index, and HDL), also response to the treatment.	[56]
	1343.0 ± 161.4 ng/mL (plasma)	Correlated with neurocognitive disturbance.	[43]
	From 1.085 (828 − 1.480) to 792 (562–1.025) ng/ml (plasma)	Has negative correlation with anti-retroviral treatment.	[57]
Leprosy	177.6 ± 62.18 ng/mL (serum)	Positively correlated with disease severity.	[58]
Visceral leishmaniasis	152.1 ± 67.86 ng/mL (serum)	Positively correlated with disease severity.	[58]
Autoimmune hepatitis	9.5 (3.3–28.8) mg/L (plasma)	Has positive correlation with disease severity and disease activity, also with treatment response.	[59]
SLE	1581 ng/ml (serum)	Diagnostic and disease activity marker for macrophage activation syndrome (MAS) in SLE.	[60]
	483.7 ± 260.8 ng/mL (serum)	Positively correlated with atherosclerosis plaque formation in SLE patients that have low cardiovascular event risk.	[61]
Lupus nephritis	67.04 ± 18.70 ng/mL (serum)	Correlated with disease severity and poor prognostic indicator.	[62]
	114.01 pg/mg (urine)	Marker for disease activity in lupus nephritis.	[63]
	2.91 ± 2.52 U/mL/mg/dL (urine)	Disease activity marker for lupus nephritis and correlated with clinical manifestation, conventional laboratory test (urea and creatinine), also renal pathology.	[64]
	22.02 (pg/mL)/(mg/dL) (urine)	Can distinguish lupus nephritis patients from SLE without nephritis also has strong correlation with activity index of renal pathology.	[65]
Glomerulonephritis	3.9 µg/ mmol (urine)	Stable marker for glomerulonephritis and can be used outside health facility also has correlation with treatment response.	[66]
Systemic sclerosis	529 ± 251 ng/mL (serum)	Potential marker for systemic sclerosis.	[67]
	984 ± 420 ng/mL (serum)	Elevated in systemic sclerosis and negatively correlated with risk for digital ulcer but positively correlated with more severe skin manifestation.	[68]
Gastric cancer	0.291–1.76 µg/mL (serum)	Diagnostic and prognostic marker in gastric cancer.	[69]

### *Soluble* CD28

**Fig. 2:**
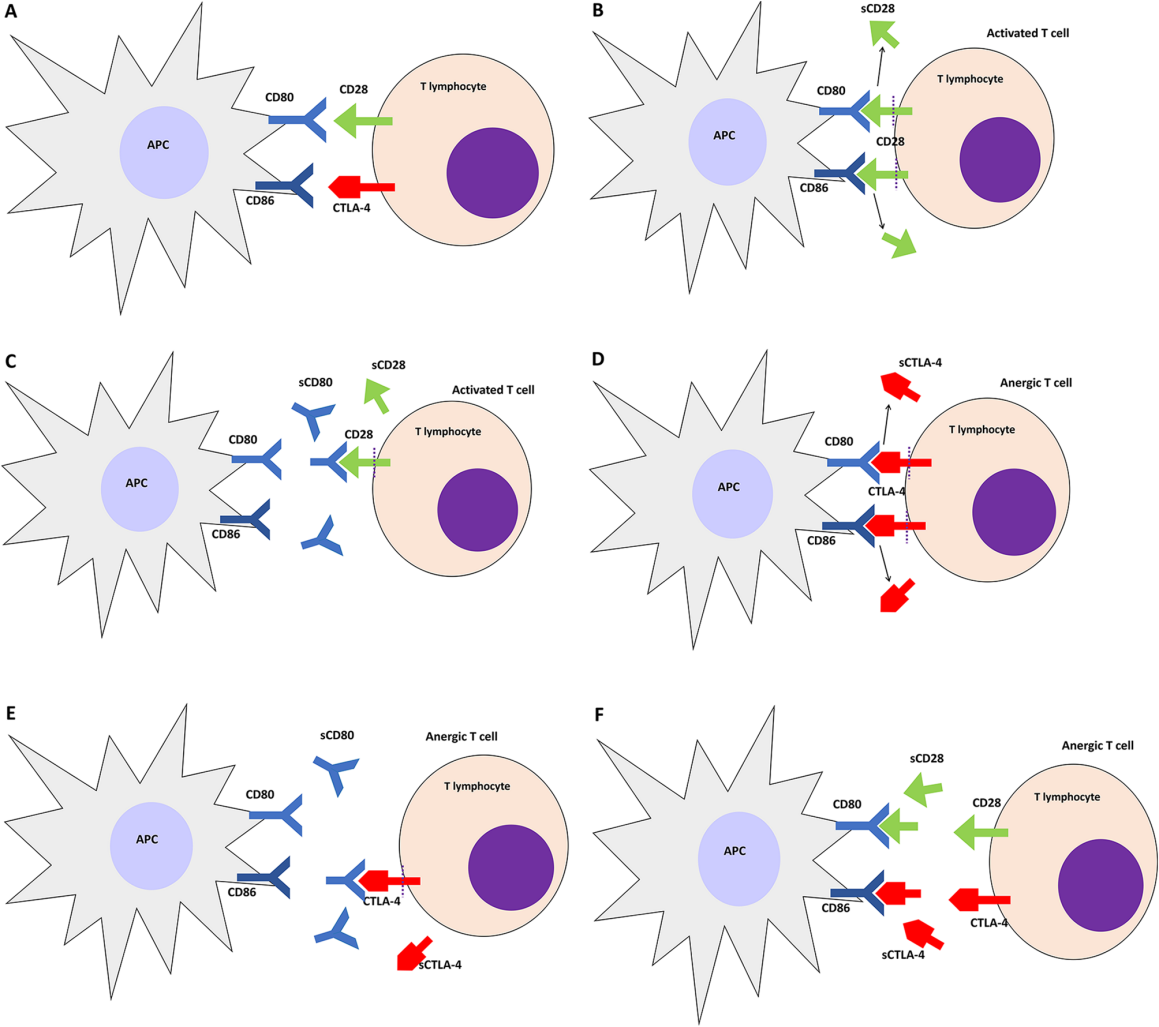
Interaction between APC receptor (CD80 and CD86) with T cell receptor (CD28 and CTLA-4). (**A**) Both CD80 and CD86 can bind to CD28 or CTLA-4. (**B**) Binding of CD80 or CD86 to CD28 will activate T cells also shedding of CD28 (become sCD28). (**C**) Binding of sCD80 to CD28 will activate T cells. (**D**) Binding of CD80 or CD86 to CTLA-4 will suppress T cells also shedding of CTLA-4 (become sCTLA-4). (**E**) Binding of sCD80 to CTLA-4 will suppress T cells. (**F**) If CD80 or CD86 bind to either sCD28 or sCTLA-4, T cells will have no response and become anergy

CD28 is a T cell surface receptor that strengthens the transcriptional effect of TCR and acts as a costimulatory receptor for naïve T cell activation [70]. CD28 is expressed in 95% CD4^+^ T cells and 50% human CD8^+^ T cells but number of CD28^+^ T cells decrease during aging and in the presence of CMV infection [71, 72]. CD28 can bind to receptors on antigen-presenting cells (APC), CD80 (B7-1), and CD86 (B7-2) for T cell activation (Fig. 2A and B). Binding of CD28 with its ligand also triggers anti-apoptosis, increases cytokine secretion, especially that of IL-2, increases cell adhesion, prevents the induction of T cell anergy, and triggers the formation of a germinal center [73]. CD28 deficiency leads to impaired T cell proliferation, changes in immunoglobulin class, germinal center formation, and impaired Th2 cell response [74, 75]. CD28 is abundant in naïve T cells but in highly differentiated T cells, number of CD28 significantly decreased [9, 13]. T cell activation occurs through its binding with membrane-bound CD28, which is followed by the shedding of membrane-bound CD28 in the plasma, referred to as soluble CD28 (sCD28). CD28 functions in T cell regulation while being expressed on the cell surface as well as in its dissolved form [76].

Soluble CD28 originates from released surface membrane receptors or alternative mRNA splicing (Fig. 2B) [31, 77]. However, recent studies using RT-PCR analysis have shown that in systemic lupus erythematosus (SLE) patients, an increase in sCD28 levels results from shedding of membrane-bound CD28 [78]. This is consistent with a study by Sun et al., who assessed sCD28 levels in Graves’ disease and found that an increase in sCD28 levels correlated with a decrease in membrane-bound CD28 [31]. In vitro, sCD28 stimulates T cell proliferation as well as IL-6 and TNF-secretion. In vivo, sCD28 serves as a marker for increased CD28 expression on T cells, indicating APC and T cell activation. Furthermore, sCD28 can compete and interfere with the interaction of CD28 or CTLA-4 with B7 (Fig. 2F) [31]. sCD28 has been found to be elevated in a variety of autoimmune diseases, including SLE and rheumatoid arthritis (RA) [76, 79] as detailed in Table 2. In addition, elevated sCD28 levels are also associated with chronic inflammatory conditions such as malignancy, chronic infection, and metabolic disorders (diabetes mellitus) (Table 2).

**Table 2:** Role of sCD28 in various chronic disease

Disease	sCD28 level (specimen)	Clinical importance	Ref
RA	1.2 ± 1 ng/mL (serum)	Has correlation with CD28 IVS3 + 17T/C allele polymorphism in T cell thus increased risk development to RA and has correlation with T/T genotype in RA patients.	[80]
	NA (serum)	Elevated in RA, correlated with treatment response but not with disease activity.	[81]
	8.8 ng/mL [7.9–11.1] (chronic RA) and 10.1 ng/mL [8.5–11.1] (acute RA) (serum)	Elevated in RA, especially in acute rather than chronic RA. Has negative correlation with anti–cyclic citrullinated peptide (anti-CCP) antibody levels and CD8^+^CD28^+^T cell count.	[76]
SLE	5.12 (3.96–6.99) ng/mL (active SLE) and 5.35 (4.21–8.90) ng/mL (inactive SLE) (plasma)	Elevated in SLE but does not have correlation with disease activity.	[82]
SLE, primary Sjögren’s syndrome (SS), and systemic sclerosis	132 ± 353 ng/ml (SLE), 290 ± 504 ng/ml (primary SS), and 83,3 ± 251 ng/ml (systemic sclerosis) (serum)	Elevated in SLE, primary SS, and systemic sclerosis. Correlated with disease activity especially in primary SS.	[78]
Grave’s disease	1.79 ± 1.52 ng/ml (plasma)	Increased in Grave’s disease, positively correlated with serum fT3, fT4, and TRAb levels, but negatively correlated with TSH level.	[31]
Myasthenia gravis	NA (serum)	Increased in myasthenia gravis and correlated with treatment response.	[83]
Neuromyelitis optica and multiple sclerosis	4.96 ± 1.90 ng/mL (neuromyelitis optica) and 4.71 ± 1.14 ng/mL (multiple sclerosis) (plasma)	Elevated in neuromyelitis optica and multiple sclerosis, slightly higher in neuromyelitis optica than multiple sclerosis. Thereis no correlation with Expanded Disability Status Scale score.	[84]
Antineutrophil Cytoplasmic Antibody (ANCA)-Associated Vasculitis (AAV)	NA (serum)	Elevated in AAV and correlated with treatment response. Potential marker for disease activity in AAV.	[85]
Asthma (adult)	1.8 (1.4–2.6) ng/mL (plasma)	Elevated in allergic asthma during corticosteroid treatment and positively correlated with serum total IgE level.	[86]
Asthma (pediatric)	0.83 (0.57–1.76) ng/mL (plasma)	Elevated in allergic asthma in pediatric during treatment but does not correlate with total IgE level.	[87]
	7.7 (6.3–10.3) ng/mL (plasma)	Highly elevated in acute asthma attack, declined after treatment, has negative correlation with peak expiratory flow rate but positive correlation with eosinophil counts and eosinophil cationic protein level. There is no correlation with total IgE level.	[88]
*Mycobacterium tuberculosis* infection	NA (serum & pleural effusion fluid)	Increased in serum and pleural effusion fluid TB infected patients, higher in pleural effusion fluid than serum.	[89]
Hepatitis B virus (HBV) infection	NA (serum)	Elevated in chronic HBV infection, correlated with ALT but not AST nor disease activity (HbeAg level).	[90]
HCV infection	≥ 1530pg/mL (serum)	Predictor marker for progression to HCC.	[91]
Gastric cancer	NA (serum)	Elevated in gastric cancer.	[92]
Breast cancer	2.65 ± 1.48 ng/mL (serum)	Elevated in breast cancer.	[93]
Uveal melanoma	NA (serum)	Increase 2.4 fold in metastasis uveal melanoma during anti-PD-1 treatment.	[94]
T2DM	19.0 (15.1–27.9) ng/mL (plasma)	Elevated in diabetic nephropathy, correlated with fasting urine albumin:creatinine ratio.	[95]
	NA (plasma)	Predictor progression to ESRD in T2DM.	[96]
	NA (serum)	Suspected to be one of risk factor of diabetic nephropathy in T2DM.	[97]
Abdominal aortic aneurism	NA (plasma)	Elevated in abdominal aortic aneurism but does not correlate with age, aneurism size, or CRP level.	[98]

Based on Table 2, sCD28 plays a role in a variety of diseases, particularly autoimmune disorders, malignancy, and chronic infections. There is a common pathogenesis in these three types of diseases in terms of chronic low-grade inflammation, which corresponds to an inflammaging condition. As a result, the authors conclude that sCD28 plays a role in inflammaging, including immunosenescence. A strong negative correlation also exists between sCD28 levels and number of CD28^+^ T cells. This demonstrates that immune system activation causes CD28 to be shed into its soluble form. Therefore, in immunosenescence (which is associated with inflammaging), an increase in sCD28 levels is likely to be observed.

### *Soluble* CD80

CD80 is a costimulatory factor expressed on the surface of activated monocytes, B cells, and dendritic cells [99]. CD80 binds to CD28 to activate T cells (Fig. 2B). CD80 expression is stimulated by APC; however, small amounts of CD80 are expressed on inactive monocytes. The soluble form of CD80, soluble CD80 (sCD80), originates from spliced mRNA or the release of cell surface CD80 receptor into the circulation [25]. The spliced form of sCD80 is expressed by inactivated monocytes and B cells [99]. CD80 prevents programmed death-ligand-1 (PD-L1) mediated immune suppression and PD-1 in tumor cells. sCD80 has the same strong ability to bind PD-L1 as that of CD80, such that sCD80 suppresses PD-L1 function. In addition, sCD80 is also able to bind CD28 and cytotoxic T lymphocyte-associated molecule-4 (CTLA-4) (Fig. 2C and E) [24, 99, 100]. When it binds to CD28, sCD80 activates T cells (Fig. 2C). However, when it binds to CTLA-4, sCD80 does not lead to either T cell suppression or activation, indicating that CTLA-4 is a receptor that functions as a decoy and does not have a biological function of T cell suppression (Fig. 2E) [24]. A study found that an increase in sCD80 levels leads to an increase in IFN-γ production by active T cells [99].

sCD80 levels were found to be > 15 µg/L in 24% of healthy individuals. However, sCD80 levels increased significantly in patients with SLE and leukemia compared to the healthy population [25]. A study found that sCD80 could prevent PD-L1 suppression and restore T cell activation by blocking interaction with PD-L1. In mice, sCD80 can slow tumor growth and trigger T cells to infiltrate tumor cells in vivo. The study concluded that sCD80 can act as a therapeutic agent to slow tumor growth [24, 100, 101]. Apart from malignancy, sCD80 also plays a role in other chronic diseases, such as minimal change disease (MCD) in adult humans, as its level in urine samples were known to increase, but not in serum. It is thought that sCD80 plays a role in the pathogenesis of MCD [102]. In addition, various studies have also found increased levels of sCD80 in numerous diseases as shown in Table 3.

**Table 3:** The role of sCD80 level in various chronic disease

Disease	sCD80 level (specimen)	Clinical importance	Ref
SLE	0.29 (0.18–0.44) ng/mL (active SLE) and 0.28 (0.16–0.40) ng/mL (inactive SLE) (plasma)	Elevated in SLE but has no correlation with disease activity.	[82]
Arthritis	15.98 ± 6.4 ng/ml (RA) 37.06 ± 8.2 ng/ml (osteoarthritis) 7.817 ± 5 ng/ml (other arthritis) (synovial fluid)	Elevated in RA, osteoarthritis (OA), and other arthritis.	[103]
RA	> 0.22 ng/ml (synovial fluid)	Elevated in synovial fluid of RA patients but not in serum.	[104]
	NA (serum)	Elevated in RA and correlated with treatment response.	[81]
Myasthenia Gravis	NA (serum)	Increased in myasthenia gravis and correlated with treatment response.	[83]
AAV	NA (serum)	Elevated in AAV and correlated with treatment response.	[85]
Asthma (pediatric)	0.36 (0.28–0.43) ng/mL (plasma)	Increased in allergic asthma and correlated with IgE level.	[87]
	0.3 (0.2–0.4) ng/mL (plasma)	Highly elevated in acute asthma attack and correlated with corticosteroid treatment response.	[88]
Nephrotic syndrome	514.01 ± 62.6 ng/mL (serum, rats) 152.48 ± 23.4 ng/mL (urine, rats)	Elevated in serum and urine nephrotic syndrome rat. Urine sCD80 level is positively correlated with total cholesterol, protein urine, and sCTLA-4 urine but negatively correlated with serum albumin level.	[105]
MCD (pediatric)	14.6 ± 30.8 ng/g creatinine (urine)	Increased in MCD and correlated with treatment response.	[106]
	524 ± 86 ng/g creatinine (urine)	Elevated in relapse MCD but not MCD in remission focal segmental glomerulosclerosis	[107]
Diabetic nephropathy	0.27 (0.20–0.41) ng/mL (plasma)	Elevated in diabetic nephropathy also, correlated with fasting urine abumin:creatinine ratio.	[95]
*Mycobacterium tuberculosis* infection	NA (pleural effusion fluid)	Elevated in pleural effusion fluid but not in serum TB patients. Has positive correlation with LDH level and lymphocyte percentage in pleural effusion fluid.	[89]
HBV infection	NA (serum)	Decreased in chronic HBV infection, protective against liver cirrhosis.	[108]
	NA (serum)	Elevated in chronic HBV infection but does not correlate with AST nor ALT level.	[90]
Alcoholic hepatitis	9 pg/mL (plasma)	Decreased in alcoholic hepatitis and correlated with disease activity, bacterial translocation, and inflammatory parameters.	[109]
HCC	NA (plasma)	Increased in HCC after trans arterial chemoembolization (TACE) but not in HCC after Lenvatinib treatment.	[110]
	≥ 82 pg/mL (plasma)	Increased in HCC post treatment with sorafenib.	[111]
Hematology malignancy	0.02–3.75 ng/ml (plasma)	Increased in chronic lymphocytic leukemia (CLL) and mantle cell lymphoma (MCL) but not in acute myeloid leukemia (AML) nor multiple myeloma (MM). Negatively correlated with prognosis, thrombocyte count, and hemoglobin level but positively correlated with leukocyte count in CLL.	[112]
Non-Hodgkin Lymphoma (NHL)	NA (serum)	Elevated NHL especially CLL and small lymphocytic lymphoma (SLL) also correlated with poor prognosis.	[113]
Soft tissue tumor	566.8 pg/mL (benign) and 609.7 pg/mL (sarcoma) (serum)	Negatively correlated with metastasis-free survival in benign soft tissue tumor and soft tissue sarcoma.	[32]
Non-small cell lung carcinoma (NSCLC)	6.32 pg/mL (serum)	Elevated in NSCLC but does not correlate with disease severity.	[114]
	65.11 pg/mL (preinvasive) and 132.06–176.76 pg/mL (invasive) (plasma)	Elevated in invasive NSCLC compared with preinvasive NSCLC also correlated with invasive disease occurrence.	[115]
Uveal melanoma	NA (serum)	Increase 1.3 fold in metastasis uveal melanoma during anti-PD-1 treatment.	[94]

Based on Table 3, it can be concluded that sCD80 level in the blood is increased in autoimmune diseases, allergies, chronic infections, and malignancies. In kidney disease, an increase in blood sCD80 level is not observed, but the levels are increased in the urine. sCD80 has so far been known as an immune checkpoint against malignancy; however, further research indicates its roles beyond that in malignancy. The authors envisage that in chronic inflammatory conditions, the elevation in sCD80 levels is caused by excess T cell activation, known as inflammaging. However, no study has reported the association of sCD80 levels with inflammaging that occurs during immunosenescence.

### *Soluble* CTLA-4

Cytotoxic T lymphocyte-associated molecule-4 or CD152 is a receptor found on T cells and plays an important role in the regulation of the immune system. CTLA-4 is homologous to CD28 and can bind to the same ligand, namely CD80/CD86 on APC (Fig. 2A) [116, 117]. CTLA-4 competes with CD28 for binding with CD80 and CD86. However, contrary to popular belief, binding of CTLA-4 to CD80/CD86 has no suppressive effect on T cells; instead, CTLA-4 acts as a decoy receptor to prevent T cell activation (Fig. 2D) [24]. CTLA-4 is strongly stimulated by activated T and B cells, and is also expressed on striated muscle cells and placental fibroblasts. In addition, 3% monocyte also express CTLA-4 on their cell surface and 20% monocytes express intracellular CTLA-4. In vitro, when stimulated by IFN-γ, monocytes secrete the soluble form of CTLA-4, soluble CTLA-4 (sCTLA-4) [117]. In vivo, sCTLA-4 is formed from alternatively spliced mRNA or comes from shedding membrane-bound CTLA-4 (Fig. 2D). Furthermore, sCTLA-4 can also be produced by T cells, especially Treg, in vitro. sCTLA-4 transcripts have been detected in lymph nodes, spleen, CD4 and CD8 T cells, B cells, and monocytes [116, 118, 119].

Several studies have found an increase in sCTLA-4 levels in autoimmune diseases such as Graves’ disease, Hashimoto’s thyroiditis, myasthenia gravis, SLE, type I DM, celiac disease, systemic sclerosis, and autoimmune pancreatitis disease. As a result, it can be concluded that sCTLA-4 levels play an important regulatory role in the immune system. sCTLA-4, on the other hand, can interfere with the interaction of CD80 or CD86 with CTLA-4, thereby blocking negative CTLA-4 signals (Fig. 2F) [116, 118–120]. Another in vitro study on melanoma cancer cells found that these cells could produce CTLA-4 and sCTLA-4, indicating a possible role of CTLA-4 and sCTLA-4 in cancer growth [33]. In addition, other studies have found that anti-CTLA-4 antibodies can bind sCTLA-4; further, sCTLA-4 has been shown to induce an antitumor response and has been suggested as an alternative therapeutic option for melanoma [99, 119]. A study in geriatric population found a positive correlation between levels of sCTLA-4 and pro-inflammatory cytokines [121]. Many studies have been conducted regarding the role of sCTLA-4 in various diseases as summarized in Table 4.

**Table 4:** The role of sCTLA-4 level in various chronic disease

Disease	sCTLA-4 level (specimen)	Clinical importance	Ref
SLE	21.6–12.3 ng/ml (serum)	Elevated in SLE but does not correlate with disease activity.	[122]
	4.05 (2.91–4.97) ng/mL (active SLE) dan 3.19 (1.73–4.67) ng/mL (non-active SLE) (plasma)	Elevated in SLE and correlated with disease activity (SLEDAI score).	[82]
	0–6326 pg/ml (median 1.044 pg/mL) (SLE) 0–4421 pg/ml (median 792.4 pg/ml) (healthy subjects) (serum)	The level is very varying in SLE and healthy subjects.	[123]
	19.58 ± 2.7 ng/ml (serum)	Elevated in SLE.	[124]
RA	NA (serum)	Elevate in RA and correlated with disease activity and treatment response.	[81]
	4.4 ng/mL (4.3–4.7) (serum)	Lower in RA compared with healthy subjects, higher in untreated RA rather than early RA patients but does not correlate with clinical condition.	[76]
	2.25 ± 0.4 ng/ml (serum)	Elevated in RA and correlated with inflammation joint count but does not correlate with laboratory test (ESR and CRP), HAQ score, and tender joint score.	[124]
Autoimmune thyroid disease (ATD)	9.8 ng/mL (serum)	Elevated in autoimmune thyroid disease (Grave’s disease and autoimmune thyroiditis) but does not correlate with clinical manifestation.	[125]
	28 to 78 ng/ml (serum)	Elevated in ATD.	[126]
Grave’s disease	7.94 ng/mL (serum)	Elevated in Grave’s disease but does not correlate with thyroid function nor Grave’s ophthalmology.	[127]
Myasthenia gravis	NA (serum)	Elevated in myasthenia gravis and correlated with treatment response.	[83]
Neuromyelitis optica and multiple sclerosis	1.86 ± 1.13 ng/mL (neuromyelitis optica) dan 1.37 ± 0.88 ng/mL (multiple sclerosis) (plasma)	Decreased in neuromyelitis optica and multiple sclerosis. There is no correlation with Expanded Disability Status Scale (EDSS) score in neuromyelitis optica and multiple sclerosis.	[84]
AAV	NA (serum)	Decreased in AAV but does not correlate with treatment response.	[85]
Spondyl-arthropathy	3.66 ± 0.3 ng/ml (serum)	Elevated in spondyloarthropathy and correlated with disease activity also CRP level.	[124]
Systemic sclerosis	> 26.5 ng/mL (serum)	Elevated in diffuse cutaneous systemic sclerosis. Positively correlated with skin fibrosis width, serum IgG level, and anti-topoisomerase I antibody level.	[128]
Psoriasis vulgaris	4.045 ± 4.466 ng/mL (serum)	Elevated in psoriasis vulgaris and correlated with disease Psoriasis Area Severity Index (PASI) score.	[129]
Celiac disease	0.0–96.4 ng/mL (serum)	Increased in untreated celiac disease, correlated with gluten intake, mucosal damage degree, also disease activity.	[130]
Autoimmune disease	6.8ng/mL (RA), 6.34ng/mL (SLE), 8.75 ng/mL (overlapping autoimmune disease) (serum)	Increased in SLE, RA, and overlapping autoimmune disease.	[131]
	NA (serum)	Increased in various autoimmune disease (autoimmune thyroid disease, celiac disease, primary biliary cirrhosis).	[132]
Asthma (adult)	2.8 (1.5–5.2) ng/mL (in non-steroid treatment), 2.9 (2.1–5.4) (in steroid treatment) (plasma)	Increased in allergic asthma and correlated with serum total IgE.	[86]
	20.2 ± 5.4 mg/L (atopic asthma), 19.2 ± 6.2 mg/L (non-atopic asthma) (serum)	Elevated in atopy and non-atopy asthma, negatively correlated with forced expiratory volume, predicted peak expiratory, and PaCO_2_, also positively correlated with lymphocytes count and disease severity.	[133]
Asthma (pediatric)	24.11 (15.19–24.33) ng/mL (plasma)	Increased in allergic asthma but does not correlate with IgE level.	[87]
	15.8 (11.3–19.2) ng/mL (plasma)	Highly elevated in acute asthma attack, correlated with corticosteroid treatment response, negatively correlated with peak expiratory flow rate.	[88]
*Mycobacterium tuberculosis* infection	NA (serum)	Elevated in serum TB patients.	[89]
Chronic HBV infection	NA (serum)	Elevated in chronic HBV infection, correlated with ALT level, but not with AST level or disease activity (HbeAg level).	[90]
	NA (serum)	Decreased in HBV infection that has progressed to liver cirrhosis.	[108]
Alcoholic hepatitis	10 pg/mL (plasma)	Decreased in alcoholic hepatitis and correlated with disease activity, bacterial translocation, and inflammatory parameters.	[109]
Abdominal aortic aneurism	NA (plasma)	Decreased in abdominal aortic aneurism but does not correlate with age, aneurism size, or CRP level.	[98]
Endometriosis	75.53 pg/mL (serum) 202.8 pg/mL (peritoneal fluid)	Increased in serum and peritoneal fluid of endometriosis stage III dan IV patients compared with stage I, II, or healthy subjects. But the level is higher in peritoneal fluid rather than in serum. Serum sCTLA-4 level has correlation with peritoneal fluid sCTLA-4 level.	[134]
Diabetic kidney disease	0.39 (0.28–0.51) ng/mL (plasma)	Decreased in diabetic kidney disease.	[95]
Nephrotic syndrome	7.70 ± 1.2 pg/mL (serum, rats) 9.64 ± 2.7 pg/mL (urine, rats)	Increased in serum and urine of nephrotic syndrome rat. Urine sCTLA-4 positively correlated with total cholesterol, protein urine, and negatively correlated with serum albumin level.	[105]
MCD (pediatric)	458 ± 652 ng/g creatinine (urine)	Increased in MCD relapse but does not correlate with treatment response.	[106]
ALL (pediatric)	132.0 ± 6208.7 ng/ml (serum)	Elevated in active B-cell ALL and positively correlated with B cell leukemia percentage also potentially can be used as progression and disease severity marker.	[135]
Breast cancer	17.8 ± 5.9 ng/mL (preganglionic involvement) 17.2 ± 5.9 ng/mL (capsular invasion) (serum)	Elevated in breast cancer with preganglionic involvement or with capsular invasion.	[93]
Gastric cancer	NA (serum)	Elevated in gastric cancer.	[92]
HCC	NA (plasma)	Elevated in HCC after trans arterial chemoembolization (TACE) but not in HCC after lenvatinib treatment.	[110]
	≥ 30.5 pg/mL (plasma)	Increased 2.64 fold in HCC after sorafenib treatment also correlated with sPD-L1 and sBTLA level.	[111]
Non-small cell lung carcinoma (NSCLC)	1.65 pg/mL (serum)	Elevated in NSCLC especially in antibody-drug conjugate group.	[114]
Malignant Melanoma	> 200 pg/mL (serum)	Elevated in malignant melanoma and correlated with best overall response (BOR) especially in immune-related stable or progressive disease, also correlated with ipilimumab treatment response.	[136]
	NA (serum)	Increased in melanoma and higher than in SLE or healthy subjects.	[33]

According to Table 4, sCTLA-4 plays a role in autoimmune disorders cancer, chronic infection, and a variety of other chronic inflammatory conditions. Because chronic inflammation is a feature of immunosenescence, the authors hypothesize that there is an increase in sCTLA-4 levels during immunosenescence. However, no research on sCTLA-4 in the context of immunosenescence in the elderly has ever been conducted.

## Conclusion

Soluble markers sCD163, sCD28, sCD80, and sCTLA-4 have promising potential to confirm the pathological condition of immunosenescence. These soluble markers were detectable in high level in the serum samples using ELISA method, thus might be and might be used to replace the cell surface receptors as immunosenescence markers. However, further research comparing their diagnostic performance with the gold standard (cell surface receptor) assay is required to make a highly accurate prediction model of immunosenensence.
